# Supplementary material for: Asymmetric participation of defenders and critics of vaccines to debates on French-speaking Twitter
Source: Sci Rep. 2020 Apr 20;10:6599. doi: 10.1038/s41598-020-62880-5 (PMC7171088; doi:10.1038/s41598-020-62880-5)
Supplement: Supplementary file 1 — Supplementary material [file 41598_2020_62880_MOESM1_ESM.pdf]

Supplementary material: Asymmetric participation of defenders and critics of vaccines to debates on French-speaking Twitter

F. Gargiulo, F. Cafiero, P. Guille-Escuret, V. Seror, J.K. Ward

1 Data collection and cleaning

The corpus of tweets was collected by an external academic service, housed by the Science–Po MediaLab in Paris, the DIME-SHS EquipEx (Excellence Facility for Survey Data Infrastructures and Methods in the Humanities and Social Sciences), a research equipment that offers researchers tools for the production or reuse of social science data and research materials. The data collection has been performed using the python based scraper, Gazouilloire [1], a tool developped by Dime Web (part of Dime-SHS) for systematic and configurable Twitter data collection through querying Twitter's official application programming interface (API) [2].

Lists of keywords and users relevant for the subject were established and used as configuration for the tool to track, following Twitter's live streaming endpoint [3] on one side, and to be collected on another hand by calling every few minutes the backsearch endpoint [4], thus allowing to collect all relevant tweets and all their metadata from up to 10 days before the collection process started and up to the end.

In order to allow user-centric analysis and to apprehend conversations, the tool also collected threads by retrieving recursively originating tweets whenever a message collected was emitted as an answer to another.

The search was not explicitly addressed to the collection of French tweets, but the query terms are in French and sufficiently specific to assure that the data volume is not exceeding the 1% limits of the streaming API.

In the following box we report the keywords, related to vaccines, on which the search was built, that have been derived by the precedent work by Jeremy Ward on the vaccine controversies in France.

[1] https://github.com/medialab/gazouilloire
[2] https://developer.twitter.com/en/docs/api-reference-index
[3] https://developer.twitter.com/en/docs/tweets/filter-realtime/overview
[4] https://developer.twitter.com/en/docs/tweets/search/overview/standard

Vaccin, Vaccins, Vaccination, vaccinations, vaccine, vaccines, Adjuvant, Adjuvante, adjuvantes, vacin, vacins, vacination, vacinations, vaxin, vaxins, vaxination, vaxinations, antivaccin, antivaccins, antivaccinaliste, antivaccinalistes, antivaccinationiste, antivaccinationniste, antivaccinationistes, antivaccinationnistes, vaxer, vaxers, vaxxer, vaxxers, antivaxer, antivaxers, antivaxxer, antivaxxers, hpv, vacc, vaccs, vacci, vaxi, vaccis, vaxis, vaccinal, vaccinals, vaccinaux, vaccinale, vaccinales, vaxinal, vaxinals, vaxinales, vaxinaux, myofasciite, myofascite, miofascite, myofaciite, myofacite, miofascite, hpvaccin, rolandsimion, revahb, sylviesimonrevelations, alisfrance, alis, alis france, myofasciite, infovaccin, infovaccins, lesfillesetlegardasil, claireseveracrebellion, initiativecitoyenne, jean-jacques-crevecoeur, crevecoeur, unisfaceauvaccin, linabmoreco, expovaccins, vaccinetruth, aluminiumetvaccins, alain-scohy, cri-vie, cri vie, questionsvaccins, professeur-joyeux, professeur joyeux, joyeux, preventionvaccin, mesvaccins, monvaccin, mavaccination, gardasil, ZOSTAVAX, DTVax, VAXIGRIP, ACT-HIB, AGRIPPAL, AVAXIM, VAQTA, Havrix, AVAXIM, BEXSERO, BOOSTRIXTETRA, Repevax, CERVARIX, DTVax, DUKORAL, ENCEPUR, ENGERIX, FLUARIX, FLUARIXTETRA, FLUENZ, GENHEVAC , PASTEUR, HAVRIX, HBVAXPRO, HEXYON, IMMUGRIP, IMOVAX POLIO, INFANRIXQUINTA, INFANRIX, INFANRIXTETRA, dTPca, Repevax, Boostrixtetra, INFLUVAC, IXIARO, RVAXPRO, MENINGITEC, MENJUGATE, MENJUGATEKIT, MENVEO, MOSQUIRIX, MenBvac, NEISVAC, NIMENRIX, PENTAVAC, PNEUMO, PNEUMOVAX, PREVENAR, PRIORIX, RABIPUR, REPEVAX, REVAXIS, ROTARIX, ROUVAX, RotaTeq, SPIROLEPT, STAMARIL, TETRAVAC, TETRAVAC-ACELLULAIRE, Boostrixtetra, TICOVAC, TWINRIX, TYAVAX, TYPHERIX, TYPHIM VI, MENINGOCOCCIQUE, VAQTA, VARILRIX, VARIVAX, VAXIGRIP, DTVax, ZOSTAVAX, Pentavalent, Hexavalent, Heptavalent, Monovalent, Trivalent, Squalene, Thiomersal, thimerosal, lnplv, Focetria, Pandemrix, Panenza, Celvapan, Humenza

After the data collection, we post–processed, with a cleaning procedure (based on Python scripts) the dataset extracted trough the APIs.

The selected queries produced false positives, above all concerning the use of the expression 'I am vaccinated against...'. This expression was above all present during the electoral period, when several Tweets expressed the immunity of the users to some politicians or some ideas. We used the topics related to diseases to remove these tweets filtering the text containing the expression 'I am vaccinated against...' (or its lexical variations) but not a disease-related word. We also removed all the lexical variations of the French expression *adulte et vacciné*, literally 'adult and vaccinated', signifying 'grown man'. We manually checked the pertinence of the first 100 most frequent tweets to the vaccine debate (deleting 2 off topic tweets from the dataset).

2 Topic selection

To select the topics in Table1 of the main text, we did not use topic modelling (like LDA) in order to avoid the over-represenataion of the extremely frequent words that result from temporally localized and spiky media events: vaccination week (*semaine de la vaccination*), information campaign on flu vaccination (*la grippe je dis non*), world day against the Sida (*journée mondiale contre le Sida*), etc. Moreover the efficiency of topic modelling methods on short texts like tweets is extremely debated. For these reason we decided to construct the set of topic on a semi–qualitative basis.
We first selected, as keywords (right column of Table1), the list of vaccine preventable diseases and of vaccines distributed in France, that we can find on this official web site[5] (with their semantic variations and abbreviations). Secondly, we grouped this set of keywords (in French as retrieved by the web page) into topics (in English). With this piece of information we constructed the classes: Seasonal, Mandatory and Other.
To complete the topic list, we performed a standard text normalization (lowering all characters, tokenization, removal of stop words) and lemmatization process on the whole corpus. We extracted from this cleaned corpus the words that more frequently appeared in the set of unique tweets (excluding retweets) fixing the lower limit to 10 repetitions. We manually selected, in this list of words, the terms that were related to awaited vaccines (Sida, Ebola, etc), converging in the class 'Awaited' and those that are connected to vaccine safety controversies (Aluminium, thimerosal, etc.), grouped in the class 'Adjuvants and Additives'.

3 Echo, polarization and media scores

The concept of echo-cambers refers to a situation where a piece of information is amplified while "bouncing" among a group of individuals. In the context of online social media, and in particular of Twitter, we can speak of an echo-chamber effect when the information flows remain trapped inside a community, namely, some pieces of information are exclusively retweeted by a defined group of users. Polarization, on the contrary, is defined as a sharp division of the opinion space, without any contact among the parts. In the case of Twitter, using the analogy of the retweet graph with an opinion similarity space, polarization can be expressed by the lack of contacts among two groups.
Finally it is important to understand the role of medias in the information flow. Since media have active profiles on Twitter, we can estimate the role of medias in a discussion according to the number of retweets of the tweets posted by media accounts.
All this information can be extracted by what we define the cross-interaction matrix among the activist groups (PRO and ANTI) and the MEDIA. To calculate this matrix we first filter the retweet network to the only nodes being

[5]https://professionnels.vaccination-info-service.fr/Aspects-pratiques/Vaccins-existants-en-France/Tableau-des-vaccins-existants-en-France

categorized as PRO, ANTI or MEDIA. We first calculate the 3×3 matrix M^{OBS} where each entry is given by the total number of links between the nodes in the categories PRO/ANTI/MEDIA. Second, we compare this matrix with a random null model: we rewire the network keeping constant the nodes degrees, similarly to what has been done in [?]. The expected number of links between the categories $C1$ and $C2$ is given by:

$$M^{EXP}_{C1,C2} = k_{C1}\frac{n_{C2}}{N_{nodes}} \tag{1}$$

Where k_{C1} is total number of outgoing links from the group $C1$.
We finally construct the cross–interaction matrix comparing the observe data and the expectations:

$$M_{C1,C2} = \frac{M^{OBS}_{C1,C2} - M^{EXP}_{C1,C2}}{M^{OBS}_{C1,C2} + M^{EXP}_{C1,C2}} \tag{2}$$

A positive value of an element of this matrix indicates that the number of connections among two classes is higher than in a random case, and viceversa. From this matrix we further define the *chamber scores*:

$$\xi_{PRO} = M_{PRO,PRO}, \quad \xi_{ANTI} = M_{ANTI,ANTI} \tag{3}$$

the *polarization score*:

$$\Pi = -M_{PRO,ANTI} - M_{ANTI,PRO} \tag{4}$$

and finally the *media score*:

$$\mu = M_{PRO,MEDIA} - M_{ANTI,MEDIA} \tag{5}$$

3.1 The global level

To better quantify the interaction level between anti/pro-vaccine activists and media we first filter the retweet graph to the only nodes belonging to one of these categories (excluding neutrals and not classified users). We introduce the group interaction matrix, M_{scores}, comparing the interaction between users in the groups with a null model where no preferences are accorded to the sources of the retweet. As we can observe in figure 1 the echo chamber effect (namely the number of intra-group connections) is extremely low for pro activist ($\xi_{PRO} = M_{PRO,PRO} = 0.097$) and moderate for activists ($\xi_{ANTI} = M_{ANTI,ANTI} = 0.49$). On the contrary the inter group connections are strongly overestimated by the null model, giving an elevate global polarization score $\Pi = -(M_{PRO,ANTI} + M_{ANTI,PRO}) = 1.88$. In general the most evident effect is the underestimation of the connections between activists (of both side) and media, giving a media score, $\mu = M_{PRO,MEDIA} + M_{ANTI,MEDIA} = 1.33$. Medias rarely retweet other than medias, and almost never retweet anti-vaccine activists.

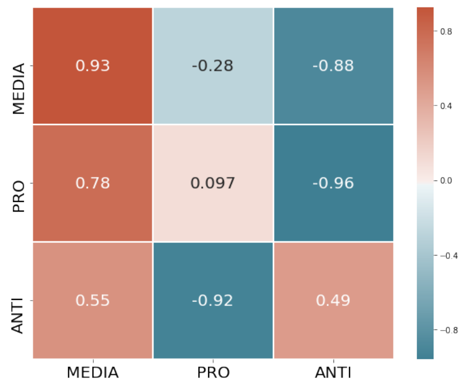

Figure 1: Cross–interaction matrix. A high (low) value of the score indicates an over(under)-estimation of the interactions by the data.

3.2 The topic level

The size of the participation of PRO/ANTI vaccine (quantifying what asserted before) and of MEDIA users in all the topics is reported in table 1. In the same table we report the results for the chamber scores ($\xi^{PRO/ANTI}$), for the the polarization score and the media score. In several topics the polarization of the debate is lower than in the case of the full retweet graph: it is the case of diseases where no particular controversies exist and that are not particularly addressed by the ANTIs (pneumococcal, tetanus, yellow fever) and where the media are central actors (like in the case of measles). Polarization scores comparable to the full graph case are reached in cases of human papilloma virus (and its vaccine) that, as we observed before is a debate where both the groups participate and media have a marginal role. Finally the highest polarization scores are reached by topics that are highly debated by the anti-vaccine activists, where the pro-activists participate without interacting with the other opponents (notice for example the high chamber score for the pro activists in the case of adjuvants). This table show that, in general, more is unbalanced the participation, namely a topic is mostly debated by one part, more the other part become self-referential.

topic	n_{Pro}	n_{Anti}	n_{Media}	ξ^{PRO}	ξ^{ANTI}	Π	μ
pneumococcal	289	36	4	0.06	0.62	1.2	-1.50
tetanus	125	35	4	0.09	0.62	1.63	-0.52
adjuvants	153	490	12	0.54	0.13	1.94	0.78
hepatitis b	72	54	11	-0.05	0.35	1.91	1.056
aids	504	66	34	0.02	0.63	1.9	1.13
yellow fever	361	8	14	-0.01	-1	1.30	1.28
polio	257	41	14	0.024	0.7	1.61	0.57
malaria	114	5	27	-0.14	-1	2	1.07
shingles	62	9	12	-0.06	0.65	2	0.99
lyme	51	18	5	0.06	0.24	2	1.33
cholera	96	10	7	0.03	-1	1.78	-0.77
hepatitis a and c	134	69	16	-0.08	0.44	1.94	1.15
vaccine ror	71	116	0	0.42	0.23	1.70	No media
ebola	102	11	24	-0.1	0.44	2	1.04
meningitis	214	104	30	-0.03	0.44	1.66	0.96
human papillomavirus	382	261	15	0.17	0.41	1.87	1.07
measles	664	25	28	-0.03	0.77	1.55	1.40
vaccine hexavalent	177	277	13	0.31	0.20	1.82	1.22
tuberculosis	125	33	16	0.03	0.52	1.77	0.95
vaccine papilloma	76	387	7	0.65	0.09	1.88	0.13
zika	92	13	31	-0.3	0.4	2	1.02
flu	818	331	47	0.09	0.53	1.78	0.48
dengue	133	10	18	-0.13	-1	1.42	1.26
whooping_cough	61	6	10	-0.24	0.85	2	-0.4
additives	46	20	2	0.13	0.54	2	-0.4

Table 1: Number of 'PRO', number of 'ANTI', number of 'MEDIA', chamber score for 'PRO' and 'ANTI', polarization score, media score.
